# Phenomenological Thermodynamics of Irreversible Processes

**DOI:** 10.3390/e20060479

**Published:** 2018-06-20

**Authors:** Yongqi Wang, Kolumban Hutter

**Affiliations:** 1 Department of Mechanical Engineering, Technische Universität Darmstadt, 64287 Darmstadt, Germany; 2 c/o Laboratory of Hydraulics, Hydrology and Glaciology, Hoenggerbergring 26, HIA 58D, ETH, CH-8092 Zurich, Switzerland

**Keywords:** Maxwell relations, Hyperbolic heat conduction, Internal variables and mesoscopic theory, Least-action and heat conduction, Mean field theory of turbulence, reaction-diffusion systems, supersonic gas ejector, Thermodynamics of gradient elasticity, Thermodynamically constrained averaging theory, Transcritical N_2_O refrigeration, Tsallis thermodynamics in 2D turbulence, Variational nonequilibrium thermodynamics

## 1. How Does Early Thermodynamics Evolve into a Rigorous Theory of Irreversibility

*Phenomenological Thermodynamics of Irreversible Processes* have its roots in the 19th century with N.L.S Carnot and his general statement in 1824 [1,2] that mechanical work can be gained from heat. J.R. Mayer in 1842 [3] formulated the equivalence principle of convertibility into heat and vice versa. He may have been the first to formulate the “First Law” of thermodynamics. It was R.E. Clausius who established the “Second Law” of thermodynamics in 1850 [4]. He phrased it in terms of the concept of “entropy” in 1865 [5] and, therefore, brought the structure of thermodynamics to a provisional closure. Nearly simultaneously, W. Thomson (Lord Kelvin) [6] had presented a different form of the Second Law in 1851. This expressed the observationally suggested and unanimously verified “principle of irreversibility” of all physical processes. The period, approximately from the mid-19th century up to the 1940s, was characterized by acquiring a deeper understanding of the principles. By introducing the concept of the absolute temperature by Lord Kelvin in 1857 [6] as a measure of coldness of a body point, the meaning of entropy as an additive monotonic quantity of a thermostatic system under adiabatic conditions (see Caratheodory 1909 [7]) demonstrates the irreversibility principle for the impossibility of “perpetua mobile”.

Substantial progress to phenomenological thermodynamics was achieved when researchers specifically addressed non-equilibrium processes of physical systems. This brought enhanced consideration of the role in physics and at scales that is above the atomic and molecular dimensions of material bodies, which could be viewed as systems continuously filled in a domain $C\in \mathbb{R}\cup {\mathbb{R}}^{+}$ and had to satisfy the balance laws of mass, momenta (linear and angular), energy, entropy, and—if electromagnetic properties would be of concern—the Maxwell equations. Due to the excess number of variables in these equations, which are generally well beyond the number of independent equations, additional relations between these variables need to be proposed, invented, or inferred from empirical information, which, together with the balance laws, will yield a system of evolution equations that will allow the unique determination of all the variables for some finite time including possible restrictions implied by the Second Law (SL). These additional relations are often characterized as being “*phenomenological*”, which suggests that they are based on less stringent information than the physical balance laws. They are often designated as “*constitutive relations*”. The union of the physical balance laws and the constitutive relations form together the so-called “*field equations*”, and the SL constrains the physical field quantities so that the reversibility requirements are obeyed. From the 1960s to 2018, most of the research activities in thermodynamics of irreversible processes have been devoted to finding solutions of the field equations including restrictions implied by the SL. In the early days of these exploitations, two paths of this endeavor have been followed to interpret the SL as a constraint condition for the processes that can be permitted by the field equations and to interpret the field equations together with the SL as a rule in order to reduce the flexibility of the constitutive relations to their simpler form. To this end, Truesdell in 1965 [8] introduced the “principle of equipresence”. We prefer to call it the “rule of equipresence” because it is no more than an instruction, which is not substantiated by any physical principle. It states that, initially, all constitutive variables are postulated to be functions of the same class of independent variables. If certain constitutive variables do not depend on some of the variables of the original class, then, according to this equipresence attitude, the deviation from the rule must be proven.

One important question in the Irreversible ThermoDynamics (ITD) method concerns how the SL is connected to the remaining physical balance laws. A priori, entropy, entropy flux, entropy production, and entropy supply are not related to any variables of the other physical balance laws. A link between the two sets is achieved by postulating that the variables of the SL, entropy, and entropy flux are constitutive relations of the class of the variables in the other physical balance laws. Similarly, the entropy source is related to the source terms of the other physical balance laws. The different forms of this postulate, which are expressed as how irreversibility is “set in action”, define the distinct forms of irreversible thermodynamics. Historically, reaching this understanding occurred from simple ad-hoc assumptions to the general understanding that was formulated above. An important step was done by Duhem in 1891 [9] who found that heat flux and entropy flux are collinear with 1/(absolute temperature) being the proportionality coefficient: ϕ = ***q***/*T*. As a rule, the external entropy supply is tacitly ignored, which corresponds to a conceptional inconsistency since the physical balance laws were thought to be valid as an open system with non-vanishing body forces, *ρ**f*** and energy supply term, *ρr*. Therefore, by mere analogy with Duhem’s relation, Truesdell in 1957 [10] postulated that the entropy supply *ρs* was connected to the heat supply *ρr* by *s = r*/*T.* Therefore, the nascent a priori estimate for the physical balance laws with the SL was:

(1) ϕ=q/T and s=r/T

in which *T* is the absolute temperature. These expressions contain an assumption that the absolute temperature, whose existence was only proven for thermostatic conditions, can also serve as a “*universal*” measure of the hotness of a body for dynamical processes.

## 2. The Clausius-Duhem Inequality as the Expression of the Second Law

By the late 1950s, the adopted SL for single constituent bodies was the inequality shown in the equation below.

(2) $$\rho \frac{d\eta }{dt}+\mathrm{div}\left(\frac{q}{T}\right)-\frac{\rho r}{T}=\rho \gamma \geq 0,$$

which had to be satisfied for all processes of materials of a given constitutive class, which satisfy the physical balance laws. More specifically, apart from Equation (2), the thermodynamic fields also satisfy the evolution equations (i.e., partial differential equations or more general functional equations) that describe all possible processes for the thermodynamic fields of the chosen material properties. This provides a different approach for open and closed systems, i.e., systems with or without external source terms.

Interpreting the concepts of open and closed systems rigorously means that the SL must be satisfactorily constrained by all field equations. However, in open systems dynamics, in which body forces and energy supplies may have arbitrary values (we assume in this discussion that no further evolution equations occur), only the mass balance equation contains no external source and must be accounted for in the exploitation of the entropy inequality.

Apart from this, the source terms of momentum and energy may take any values. In particular, the body force ***f*** and the energy supply *r* may at any material point and any time take values such that they outbalance all other terms in the remainder of these equations. Moreover, the energy equation may be used to eliminate the source term in the Clausius-Duhem inequality because of Equation (1). This leads to the energy and entropy equations.

(3) $$\rho \dot{\varepsilon }=-\mathrm{div}\;q+\mathrm{tr}\left(\sigma \;D\right)+\rho r\;,\;\rho \dot{\eta }=-\mathrm{div}\;\left(\frac{q}{T}\right)+\frac{\rho r}{T}+\rho \gamma ,\;\left(\gamma \geq 0\right)$$

It also leads to the dissipation inequality below.

(4) $$\;\rho \left(\dot{\varepsilon }-\;T\dot{\eta }\right)-\mathrm{tr}\left(\sigma \;D\right)+\frac{q.\mathrm{grad}\;T}{T}\geq 0\;.$$

in which the various symbols are defined in the table “*List of symbols*”. In its exploitation, the momentum and energy balance do not act as constraints since they have freely assignable source terms, but the balance of mass needs to be accounted for since it has no source term. In short, introducing constitutive relations for the fields *F* of the form F=f(ρ, D, grad T=g, T) for $F\in \left(\varepsilon ,\;\psi ,\;\eta ,\;\sigma^{E},\;p\;,q\right),\;\mathrm{where}\;\psi =\varepsilon -T\eta$ is the Helmholtz free energy and $\sigma =\sigma^{E}-$p**1**, in which $\sigma^{E}$ is the extra-stress and p the pressure, it is straightforward to show that Equation (4) takes the form below.

(5) $$-\rho \left\{-\frac{\partial \psi }{\partial \rho }\rho -\frac{p}{\rho }\right\}\mathrm{div}\;v+\left\{\frac{\partial \psi }{\partial T}+\eta \right\}\dot{T}+\frac{\partial \psi }{\partial D}\cdot \dot{D}+\frac{\partial \psi }{\partial g}\cdot \dot{g}+\sigma^{E}\cdot D-\frac{q.g}{T}\geq 0,$$

in which the first term on the left-hand side the balance of mass $\mathrm{div}\;v\;=\;-\left(\dot{\rho }/\rho \right)$ has been employed. It is readily observed that $\dot{T},\;\dot{D},\;\mathrm{and}\;\dot{g}$ in Equation (5) can take arbitrary values without violating the balance laws of momentum and energy (since the external source terms have been freely assigned). Due to the linearity of Equation (5) in $\dot{T},\;\dot{D},\;\mathrm{and}\;\dot{g}$ the coefficients of these terms must, therefore, vanish. This leads to the statements below.

- The free energy is neither a function of D nor of g: ∂ψ/∂D=0, ∂ψ/∂g=0.
- The free energy is a potential for $-\eta \;\mathrm{and}\;p/\rho^{2}\;$, i.e., −η=∂ψ/∂Tand $p/\rho^{2\;}$= ∂ψ/∂ρ. This implies
  (6) $$\;\left(-\eta ,\;\frac{p}{\rho^{2}}\right)=\nabla_{T,\rho }\psi \left(T,\;\rho \right)$$
  and illustrates this potential relation. $\left(-\eta \;\mathrm{and}\;p/\rho^{2}\right)$ are called to form canonical pairs to *T* and ρ.
- These results lead quite naturally to the Gibbs relation.
  (7) $$d\eta =\;\frac{1}{T}\left\{d\varepsilon +pd\left(\frac{1}{\rho }\right)\right\}.$$

The Coleman-Noll axioms in 1963 [11] have, in this case, proven the form of the Gibbs relation. We emphasize, however, that, in the context of this thermodynamic approach, its form has to be derived. Nevertheless, some authors start with an axiomatically postulated Gibbs relation, which is shown in this volume. These details are given in any book on phenomenological thermodynamics (e.g., Hutter & Jöhnk [12], Hutter & Wang [13,14], Truesdell [15], Eringen [16], and others).

This scheme of exploitation of the SL has been given by Coleman & Noll [17] and has been used commonly by many authors dealing with establishing thermodynamic restrictions implied by the SL. Critical minds in physics may question the supposition that the source terms can be freely assigned even though mathematically this is compulsory for the inferences that one draws from it. What it requires is that the material specimen can be brought into an environment in our universe in which any values of the body force and energy supply can be generated so that the specimen may be physically melted or destroyed. The introduction of source terms in balance expressions for quantities expressing some esoteric internal properties of material behavior (e.g., in descriptions of turbulence, in which balance relations for averages of correlation terms of products of velocity fluctuations with temperature fluctuations etc. are formulated, or in polycrystalline materials when stress induced anisotropies are in focus), are described by balance laws and source terms are introduced in these laws with the only purpose to generate a variable—quasi as “*deus ex machina*” (“Deus ex machina”; is a Latin calque from Greek ἀπὸ μηχανῆς θεός, meaning “god from the machine”. The term was coined from the conventions of Greek tragedy where a machine is used to bring actors playing gods onto the stage. In literary texts, it means that in an argument that cannot rationally be closed, an ad-hoc assumption is introduced, which allows closure of the argument. It is very much similar to an axiom in mathematics). This is conducted in order to ignore these equations as constraints in the exploitation of the dissipation inequality.

## 3. The Müller-Liu Procedure of the Exploitation of the Second Law

The a priori estimates (1) of the entropy flux and entropy supply that led to the Clausius-Duhem inequality were questioned first by Müller in his Ph. D dissertation in 1966 [18], in his ARMA article in 1967 [19], and in his habilitation thesis at TU Aachen, which was published in ARMA in 1971 [20]. These articles attempt in several steps to amend fundamental weaknesses of classical ITPs. Within the Coleman-Noll scheme of the exploitation of the SL, the Gibbs equation turns out to be a unique functional relation in both static and dynamic processes. As a consequence, entropy and pressure in a dynamic process do not depend on the flux terms of the physical balance laws including viscous stress and heat flux vector. They follow the dynamic processes. The same holds true for all other field variables, which are derived via Legendre transformations from alternative thermodynamic potentials (free enthalpy, Gibbs free energy; recall the Maxwell relations). Therefore, one adds the flux terms and exploits the dissipation inequality by enlarging the constitutive dependences by the flux terms.

Müller, in his 1967 [19] article takes a step in relaxing the Duhem expression for the entropy flux ${\left(1\right)}_{1}$. This generalization is simply obtained by requesting ϕ to be a constitutive quantity of the same class as the other constitutive variables. This paper has been of transitional significance because it brought clarity in that the entropy flux and heat flux vectors did not have to be affine (as is the case for ${\left(1\right)}_{1}$). In mixtures, such a co-linearity did not exist (see e.g., Müller [21], Hutter & Jöhnk [12], Chap. 7). The decisive basic assumption for the exploitation of the SL was given in the 1971 ARMA paper [19]. This entropy principle is quoted and listed in Hutter & Jöhnk [12], Chap. 5, p. 207.

The Clausius-Duhem inequality includes the adoption of the absolute (Kelvin) temperature as a measure of the hotness or coldness of a material particle of a body. It has been shown to be a useful concept in thermostatic processes yet has not been proven but simply conjectured to be the coldness measure in thermodynamic processes.

### Entropy Priciple (EP) (by Müller in 1971 [19])

*1.*  *In every material body, there exists an additive quantity, the specific entropy η, which obeys a balance equation.*
  (8) $$\rho \frac{d\eta }{dt}=\;-div\;\phi +\rho s+\rho \gamma ,$$
  *in which ϕ is the entropy flux, s is the specific entropy supply, and γ is the specific entropy production.*
*2.*  *The specific entropy η and the entropy flux* ϕ *are material quantities for which, according to the rule of equipresence, the same material laws hold as for the remaining constitutive quantities.*
*3.*  *The entropy production must be a non-negative quantity.*
   γ≥0
  *i.e., for all solutions of the field equations (these are the balance equations plus the constitutive relations together).*
*4.*  *The supply terms, which appear in the balance equations, cannot influence the material behavior.*
*5.*  *There exist special material surfaces, the so-called ideal walls between two continual walls, across which the empirical temperature and the tangential velocity are continuous.*

Special points, which this EP expresses, are outlined below.

- Entropy is an additive quantity for static processes. In modern studies, however, entropy is often postulated to be non-additive. In these cases, the above EP must be changed.
- Thermodynamic processes for a certain material class are in the context of the applicability of the equipresence rule for all solutions of the field equations.
- External supply terms cannot influence the material behavior. This means that influences of the SL to the constitutive relations can be deduced from the supply free physical balance laws and paired with the supply free entropy balance. This means that the EP applies for closed systems thermodynamics. The same inferences can be drawn for open systems thermodynamics, according to item 4 of the EP since this statement means that the entropy supply must, at most, depend on the remaining supply terms of the physical balance laws.
- In item 5 of the EP, the concept of an ideal wall is introduced. This concept is needed because a quantity that may replace the absolute temperature is not incorporated in this SL (as it is in the Clausius-Duhem inequality). Using temperature as a variable requires an empirical measure θ (in instruments a pressure, volume expansion or electrical conductivity etc., monotonically changing with our sensation of hotness/coldness). Similar to a Duhem relation, ϕ=q/T, can be proven by the EP. Then this divisor *T* should be a universal functional, e.g., $T=T\left(\theta ,\dot{\theta },\ldots \right)$ that is materially independent and reduces to the absolute temperature under thermostatic processes. Item 5 in the EP achieves this result.

The formulation of the above EP serves as a guideline for a stringent mathematical instruction to a wealth of thermodynamic processes of continuous media of a certain material class. Its most serious restriction is likely the postulate that entropy is an additive quantity. The latest research studies by physicists, however, concentrate on non-additive formulations of entropy under dynamic processes, which points at necessary revisions of the above EP.

Liu’s theorem [22] is technically relevant for the application of the EP. This is a special case of a much broader theorem well known in operations research, which is originally due to Farkas [23] and Minkowski [24]. It states that the entropy inequality, subject to a number of field equations, is equivalent to the satisfaction of the statement below.

(9) $$\left\{\mathrm{entropy}\;\mathrm{balance}\right\}-\Sigma_{i\;}\Lambda_{i}\cdot {\left\{\mathrm{field}\;\mathrm{equation}\right\}}_{i}\geq 0\;(\mathrm{EEI})$$

in which $\Lambda_{i}$ is the Lagrange parameter associated with the field equation and the label i. The dot · denotes scalar multiplication. In the above form (EEI), determining the Lagrange parameters is part of the exploitation of EEI, which is given for a closed system. In a subsequent paper, Liu in 1973 [25] made the Lagrangean concept applicable to open systems by extending (EEI) to balance laws with external source terms. If $s_{i}$ is the source term of the ${\mathrm{ith}}^{}$ balance law and *s* is the entropy supply, then we may write the equation below.

(10) $$s=\Sigma_{i}\lambda_{i}s_{i}\;,$$

where $\lambda_{i}$ are factors of proportionality not dependent on $s_{k}\;\left(k=1,\;2,\ldots \right)$. Equation (10) is a generalization of ${\left(1\right)}_{2}$ for a conceptual generalization without the absolute temperature as a primitive concept. It is not a constitutive concept but a statement that the external supply terms of the theory should not affect the implication of the SL. For such an open system, (EEI) is replaced by the formula below.

(11) $$\left\{\mathrm{entropy}\;\mathrm{balance}\right\}-\Sigma_{i}\Lambda_{i}\cdot {\left\{\mathrm{field}\;\mathrm{equation}\right\}}_{i}-\Sigma_{i}\lambda_{i}\cdot {\left\{\mathrm{ext}.\mathrm{source}\;\mathrm{terms}\right\}}_{i}\geq 0.$$

For extensive applications, see Müller [26,27], Hutter [28], Liu [29], Hutter & Wang [14], and many others.

## 4. The Use of Nonlinear Entropic Functions

An alternative approach to ITD, validity for processes distant from thermodynamic equilibrium is what some specialists of nonlinear dynamics call extended thermodynamics (ETD). They introduce concepts of generalized entropies, which, in equilibrium, must be reduced to the Boltzmann-Gibbs (BG) entropy because Boltzmann [30] and Gibbs [31] statistics are the basis for the thermodynamic equilibrium. Reference to a SL is not made when generalizing this extended entropy concept. However, it is stated that this kind of entropy concept is compulsory to be able to describe nonlinear processes. We start with classical BG-statistics and seek extensions of it by nonlinear adjustment [32]. Haken [33,34] developed and summarized cooperative dynamics in physical systems with a hierarchical structure. He introduced the so-called slaving principle in which a certain mode can reach a superior value compared to others, which presents itself as an ideal order parameter of a complex system. This procedure was apt to introduce statistical descriptions of nonlinear thermodynamic systems. A number of nonlinear entropic functions have been introduced and are appropriate for particular applications, e.g., Kullback-Leibler entropy, Normalized entropy, Shannon entropy, Rényi entropy, Tsallis entropy, Kolmogorov-Sinai entropy, Escort Entropy and others, see Beck & Schlögel [35] and Teisseyre & Majewski [36]. 

To introduce nonlinear entropic functionalism, we start with the BG entropy of statistical mechanics $\eta_{BG}=-k\sum_{i=1}^{N}p_{i}\log_{e}p_{i}$ where the $p_{i}$’s denote the microscopic probabilities of the system comprised of N subsystems. k is the Boltzmann constant. If all subsystems carry the same probabilities, we have $p_{i}=1/N$ and $\eta_{BG}=k{\;\log}_{e}N\;\mathrm{or}\;N\left(\eta_{BG}\right)=\exp\left(\eta_{BG}/k\right)\;$. For two probabilistic independent sub-systems A&B with $N_{A\;}\mathrm{and}\;N_{B\;}$ in contact, one has $p_{ij}^{A+B}=\;p_{i}^{A}$+$p_{j}^{B},\;\forall \;i,j\in \left(1,\ldots ,N\right)$. It is easy to show that $\eta_{BG}\left(A+B\right)=\eta_{BG}\left(A\right)+\eta_{BG}\left(B\right).$ Therefore, the BG entropy is additive. Moreover, it is also easy to show the linear initial value problem.

(12) $$\frac{dN}{d\eta_{BG}}\;=\;\frac{\;1}{k}N,\;N\left(0\right)=1,\;\mathrm{solves}\;\mathrm{as}\;N\left(\eta_{BG}\right)=\exp\left(\frac{\eta_{BG}}{k}\right).$$

The Tsallis entropy [37] is obtained from the simplest generalization of this initial value problem.

(13) $$\;\frac{dN}{d\eta_{q}}=\;\frac{1}{k}N^{q},\;N\left(0\right)=1,$$

where q∈ℝ and *k* is a constant, possibly different from the Boltzmann *k*, while $\eta_{q}$ is the Tsallis Entropy. The solution of Equation (13) subject to the condition that for N=1, we have $\eta_{q}=0,$ which is shown below.

(14) $$N\left(\eta_{q}\right)=\;{\left\{1+\left(1-q\right)\frac{\eta_{q}}{k}\right\}}^{1/\left(1-q\right)}\;↔\;\eta_{q}=\frac{k}{1-q}\left(N^{1-q}-1\right).$$

Comparing these expressions with the corresponding BG-results, these formulae suggest the introduction of the *q*-exponential and *q*-logarithmic functions.

(15) $$e_{q}\left(x\right)={\left(1+\left(1-q\right)x\right)}^{1/\left(1-q\right)}\;↔{\;\ln}_{q}\left(x\right)=\frac{1}{1-q}\;\left(x^{1-q}-1\right),$$

which for *q* = 1 reduce the classical exponential and logarithmic functions (replace $\frac{1}{1\;-\;q}$ by *n* and take the limit as *n* moves to infinity).

Motivated by the above generalization, the $\eta_{q}$-expression for equal probabilities among the sub-systems can now be postulated to have the form $\eta_{q}\left(N\right)=k\ln_{q}\left(N\right)$ and with $p=\frac{1}{N},\eta_{q}\left(p\right)=k{\;\ln}_{q}\left(\frac{1}{p}\right).$ Adding over all subsystems *i =* 1, …, *N* yields the equation below.

(16) $$\eta_{q}\left(N\right)=\sum_{i=1}^{N}p_{i}\ln_{q}\left(\frac{1}{p_{i}}\right)=\frac{k}{1-q}\sum_{i=1}^{N}p_{i}\left(p_{i}^{q-1}\right)=\frac{k}{1-q}\left(1-\sum_{i=1}^{N}p_{i}{}^{q}\right),$$

which for *q* = 1 shows that $\eta_{1}=\eta_{BG}$. Expression (16) is the *q-entropy* or the *Tsallis entropy*, which is a “natural” nonlinear generalization into non-equilibrium thermodynamics. This equation is shown below.

(17) $$q<1\;↔\;p_{i}^{q}>p_{i},\;q=1\;↔\;p_{i}^{q}=p_{i},\;q>1\;↔\;p_{i}^{q}<p_{i}\;.$$

Furthermore, with the use of formula (15), it is straightforward to prove the equation below.

(18) $$\;\eta_{q}\left(A+B\right)=\eta_{q}\left(A\right)+\eta_{q}\left(B\right)+\frac{\left(1-q\right)}{k}\eta_{q}\left(A\right)\eta_{q}\left(B\right),$$

which, when paired with (17), implies the following.

(19) $$q<1:\eta_{q}\left(A+B\right)>\eta_{q}\left(A\right)+\eta_{q}\left(B\right);\;q>1:\;\eta_{q}\left(A+B\right)<\eta_{q}\left(A\right)+\eta_{q}\left(B\right),\;$$

recovering equality and additivity for *q* = 1.

## 5. The 12 Contributions Published in This Special Issue

These are discussed in the order of appearance in this special issue of *Entropy*.

J. Weberszpil & W. Chen [38] discuss “*Generalized Maxwell Relations in Thermodynamics with Metric Derivatives*”. These are equations, which establish relations between pressure, specific volume, entropy, temperature, and thermodynamic potentials such as internal energy, Helmholtz free energy, enthalpy, and Gibbs free energy. These potentials do, for a certain constitutive class, only depend on variables that “*survive*” in thermodynamic equilibrium and emerge from the exploitation of the Gibbs relation using the appropriate Legendre transformations. For the classical theory, see Hutter & Wang [14].

The obtained different Gibbs and Maxwell relations are also postulated to hold in extended thermodynamics with generalized entropic forms, but, to this end, it is convenient to replace the classical derivatives with respect to (the newly defined) entropy, specific volume, temperature, and pressure by “stretched” derivatives (our denotation). Depending upon this “*stretching*”*,* different forms of dynamic entropies and associated Maxwell relations emerge. The new differentials are called metric, q-total, and conformable. The deformed Maxwell relations take in each case their separate form. Technically, a large part of the paper is devoted the mathematical developments of the new calculus.

S. Croquet, S. Pocet & Z. Aidoun [39] present in “*Thermodynamic Modelling of Supersonic Gas Ejector with Droplets*”, a study of determination of the entrainment ratio and double choke limiting pressure of supersonic ejectors within heat driven refrigeration cycles with and without droplet injection at the constant area section of the device. The aim is to compare the computational performance of the device with experimental data. The basic thermodynamic formulation is well known for gas dynamics interacting with droplets. Therefore, the aims and goals of this carefully written paper are in its explicit comparison between the computed and measured output data for well-described input conditions.

P. W. Egolf & K. Hutter [40] in “*The Mean Field Theories of Magnetism and Turbulence*” studied the turbulence as a cooperative and critical phenomenon with a critical Re-number at its onset. Critical or cooperative phenomena are distinctive features in physics and their analogies demonstrate near or perfect universal behavior. The mean field theories of magnetism and turbulence demonstrate the universal behavior. For instance, the paramagnetic-ferromagnetic phase transition manifests itself as a paramagnetic region with disordered magnetization that incorporates patches of Weiss domains. Above the critical temperature, only the paramagnetic phase exists. As the temperature is ignored, the oriented patches grow until they fill the entire material space. In a turbulent system, the lowest-order phase with the highest symmetry properties is laminar flow and the highest-order phase, in this case, is an infinite Re-number turbulent flow. Therefore, in a medium Re-number flow, we may be able to distinguish subdomains of calm laminar streaks from regions of turbulent activity.

In detail, it is shown that symmetry braking occurs in both systems with analogous critical behavior and that mean field theories of paramagnetic to ferromagnetic phase transitions (known) show stunning correspondences to the mean field theory of turbulence (new). These facts are subtleties, which hold between the two systems beyond the thermodynamic correspondences in the background.

S.-Y. Li & B.-Y. Cao [41] address in the “*Entropic Constitutive Relations and Modeling for Fourier and Hyperbolic Heat Conductions*” the problem to replace these equations, which is expressed as PDE’s for the absolute temperature by corresponding equations for the entropy. Their basic PDEs are the Fourier and Cattaneo-Vernotte (CV) models and they employ in their transformation the structure of the SL in the form of classical irreversible thermodynamics (CIT) and extended irreversible thermodynamics (EIT). The entropic evolution equations are formulated for both the entropy, its flux, and separately for both the Fourier and CV heat flow problems. Correlations between these Boltzmann-Gibbs-Shannon (BGS) entropy formulas and statistical mechanics establish expressions for the BGS-entropies and the CV-entropies in terms of the relaxation time of the CV heat equation.

Z. Zhang, Y. Hou & F. Kulacki [42] studied “*Energetic and Exergetic Analysis of a Transcritical* $N_{2}O$ *Refrigeration Cycle with an Expander*” in an attempt to replace the chlorofluorocarbon and hydrofluorocarbon refrigerants with environmentally less damaging alternatives. The nitrous oxygen $N_{2}$O as a refrigerant is not yet fully explored. Its exergetic performance is analyzed and compared with that of ${\mathrm{CO}}_{2}$. The results are represented in the graphs.

C. Pappenfuss, & W. Muschik [43] “*Macroscopic Internal Variables and Mesoscopic Theory: A Comparison Considering Liquid Crystals*”. Thermodynamics of complex materials is treated in two different forms.

(i) The introduction of additional space state variables consists of position and time plus additional variables accounting for the internal structure of the complex material, e.g., porosity in porous media. This work modeled solid mechanical structures, ice sheets of the Earth, and more. Internal variables may also be incorporated in a material theory with no other explicit definition than equations that are written down for them. For instance, the climate relevant gases of the air trapped in the air bubbles of glaciers and ice sheets serve as parameters for the ice fluidity and ice age in climate relevant studies [44].
(ii) The second approach is the so-called mesoscopic method whose domain of the field quantities (x, t) is enlarged by mesoscopic quantities, which gives rise to the mesoscopic space ${\mathbb{R}}_{3}^{3}\times {\mathbb{R}}_{t}\times M$ in which *M* consists of variables defining the complex local behavior of the material. This procedure often entails partial or full embedding of statistical components in the theory.

The aim of the paper is to thoroughly discuss the connection between the macroscopic theory involving internal variables depending on space and time with the mesoscopic theory formulated within the context of the mesoscopic state space. Both classical irreversible and extended thermodynamics can be used as vehicles to derive restrictions implied by the irreversible principle. The authors use the Duhem-Truesdell relations (Equation (1)) to derive the implications of the dissipation principle.

As their illustrative examples demonstrate the conceptual observations, they use the various levels of the liquid crystal theories either by basing them on director concepts [45,46] and anisotropy or conformation tensors (emphasizing the works of the TU-Berlin school). Further applications of the mesoscopic theory are devoted to damage in solids by micro-cracks and to dipolar media (i.e., fluid crystals without head-tail symmetry). Para-ferromagnetic materials has second order phase transformations and suspensions of fibers in fluids. The literature in physics on such complex materials is overwhelming.

P. W. Egolf & K. Hutter [47] “*Tsallis Extended Thermodynamics Applied to 2-d Turbulence: Lévy Statistics and q-Fractional Kraichnanian Energy and Enstrophy Spectra*”. This article combines thorough reviews of Boltzmann-Gibbs (BG) and Tsallis extended thermodynamics to 2-d turbulence. On the basis of the profound studies of Kraichnan in which the force-free momentum equations and the energy and entropy expressions are discretized by spectral (Fourier) Ritz-Galerkin projections, which are truncated by eliminating the components with the smallest wavelengths (corresponding to the Kolmogorov scales). Among wavelengths larger than the size of the domain, it is shown that the turbulence of incompressible Euler fluids conserve the overall energy and enstrophy. These properties remain invariant under Ritz-Galerkin projections. However, in the context of BG statistical thermodynamics, this projection leads to a loss of the essential features of low wavenumber turbulence.

On the other hand, it is surprising to observe that the entropy functional and the partition function define “*universality classes*”, which show identical “*critical exponents*”. Otherwise stated, a system close to its criticality exhibits a kind of universal behavior that is not determined by the specific microscopic interactions at the sub grid dimension (see the article by Egolf & Hutter [40] on the mean field theory of magnetism and turbulence).

A statistical theory different from BG statistics and an extended entropy concept is, therefore, needed to cope with these facts. Among the available nonlinear entropy concepts, the Tsallis entropy turns out to be a fortunate improvement over the BG entropy. By nonlinearly extending the ordinary differential equation, which led Boltzmann to the exponential and logarithmic functions, naturally led to the non-additivity of the Tsallis entropy, which correctly describes sub-dispersive, Brown, and super-dispersive diffusion with the power exponents *q* < 5/3, *q* = 5/3 and *q* > 5/3, respectively. In addition, there exists a convincing relation between Lévy statistics and Tsallis’ extended thermodynamics and the fractional generalization of Kraichnan’s spectra leads to the well verified Kolmogorov-Oboukov energy spectrum of $k^{-5/3}$. Moreover, by introducing the fractal behavior of the scaling law among the eddies of the various sizes, the evident self-similarity of the eddy cascade is explained. From the theory, in a natural manner, a generalized temperature of turbulence is revealed that shows that small-scale eddies are in “thermodynamic equilibrium” (after BG) while eddies above a threshold wave length are non-equilibrium “objects”. This different behavior is characterized by different power-law exponents of the energy density spectrum. The low-wave-number entropic part shows the Kolmogorov-Oboukov value (see above). The high-wave number energy part of the spectrum shows the equilibrium power law derived by Kraichnan being k^−3^.

K. Weinberg, M. Werner, & D. A. Anders [48] “*Chemo-Mechanical Model of Diffusion in Reactive Systems*”. The aim of this work is to model solid mechanical structures, which undergo large elastic and inelastic deformations coupled with diffusion processes and chemical reactions. The authors propose a nonlinear chemo-mechanical model of diffusion in reactive multi-component systems, which are framed in the context of non-equilibrium thermodynamics of de Groot-Mazur [49]. They describe the theoretical background of this kind of mixture formulation (of class I) and present numerical results obtained with the finite element technique using NURBS of samples of binary and ternary reaction diffusion systems with and without large elastic deformation.

Mathematically, the deformation, referred to the reference configuration and modelled for the mixture as a whole, and deformation behavior are based on the right Cauchy-Green deformation tensor. Balances of the diffusing and reacting components are formulated in terms of their concentrations with the diffusion fluxes expressed in terms of the mobility tensor and the chemical potentials. The latter are described by the Helmholtz free energy that is additively composed of elastic, configurational, and interfacial components. Of special interest is the section on the dynamics of the chemical reactions, the results of which enter the diffusion equations as production terms. In its last part, the manuscript is devoted to some fascinating but brief computational studies of ternary structural multi-component gadgets.

F. Gay-Balmaz & H. A. Yoshimura [50] “*A Variational Formulation of Non-equilibrium Thermodynamics for Discrete Open Systems with Mass and Heat Transfer*”. This contribution presents a variational formulation for non-equilibrium thermodynamics of discrete systems with a number of ports through which heat and mass can be exchanged with the environment. It combines an overall energy balance (Fist Law) and entropy balance (Second Law) in the context of a general varied formulation with time dependent nonlinear non-holonomic constraints and time dependent Lagrangean. The theoretical structure is based on Stueckelberg & Scheurer’s and the authors’ precursory work in the Lagrange-d’Alembert principle for non-holonomic mechanics is extended to encode the internal entropy production due to irreversible processes into a class of nonlinear thermodynamic constraints.

The varied formulation of discrete open systems is mathematically and consecutively structured with prerequisites, conjectures, and proofs of statements not in an exclusive mathematical jargon but still requiring some versatility of its language. The developments are interspersed with remarks that mention the physical situation to emphasize the results and to emphasize connections with related situations. Several examples are used to demonstrate the applicability of the concepts.

(i) Filling an insulated tank with a pressurized gas,
(ii) Flowing fluid carrying kinetic energy into a piston device,
(iii) Diffusion between two components,
(iv) Diffusion through a series array of membranes,
(v) Diffusion through a parallel array of membranes,
(vi) Heat conduction and diffusion between two compartments,
(vii) Heat conduction and diffusion through a composite membrane.

H. Alber, C. Broese, C. Tsakmakis & D Beskos [51] “*Non-Conventional Thermodynamics and Models of Gradient Elasticity*”. This “non-conventional thermodynamic axiomatics” is suitable for gradient elasticity models. Additionally, it is based on ideas put forward by Toupin [52], Mindlin [53], Mindlin & Eshel [54], Dunn & Serrin [11], and Maugin [55]. To sketch the ideas of the non-conventionalism, let us start with the energy equation and the Clausius-Duhem inequality.

(20) $$w_{st}-\dot{\psi }-T\dot{\eta }-\eta \dot{T}-q_{i,i}\;=\;0,\;\gamma \cdot \dot{\eta }+\;{\left(\frac{q_{i}}{T}\right)}_{i}\geq 0,$$

in which $w_{st}$ is the power of working ψ the Helmholtz free energy, η the entropy, *T* the absolute temperature, and $q_{i}$ the heat flux vector. It is well known that, for classical thermodynamics, the absolute temperature and entropy only depend on equilibrium variables (e.g., on strain but not on its gradient). If we denote these equilibrium variables by $v_{I},\;I=1,\ldots ,\;N_{I}$, then the analogue of the free energy is assumed to have the form below.

(21) $$\psi =\psi \left(v_{I},{\tilde{\zeta }}_{J},T\right),$$

where ${\tilde{\zeta }}_{J}$ are the components of the time and space derivatives of *T*. Note, ψ does not involve time and space derivatives of *T.* If the strains $\varepsilon_{ij}$ and their gradients $\varepsilon_{ij,k}$ are elements of the state space, they engender higher order stresses and additional terms in the stress power with the latter being defined as ${\bar{w}}_{st}$. It is similar to the heat flux ${\bar{q}}_{i}$. Therefore, the First Law, expressed in terms of ψ now takes the form below.

(22) $${\bar{w}}_{st}-\dot{\psi }-T\dot{\eta }-\eta \dot{T}-{\bar{q}}_{i,i}=0$$

Defining

(23) $$w_{st}^{\prime }\cdot w_{st}-{\bar{w}}_{st},\;q_{i}^{\prime }\cdot q_{i}-{\bar{q}}_{i},$$

combining (20) with (22) and (23) yields $w_{st}^{\prime }=\;q_{i,i}^{\prime }$ and with (25)

(24) $$w_{st}^{\prime }=\sum_{k=1}^{N}{\left(w_{st}^{\prime }\right)}_{k},\;q_{i}^{\prime }=\sum_{k=1}^{N}{\left(q_{i}^{\prime }\right)}_{k}.$$

Lastly, by postulating that energy transfers into mechanical power states, we obtain the equation below.

(25) $${\left(w_{st}^{\prime }\right)}_{k}=\;{\left[{\left(q_{i}^{\prime }\right)}_{k}\right]}_{,i}\;,\;k=1,\ldots ,\;N.$$

To complete the theory, constitutive relations must be established for ${\left(w_{st}^{\prime }\right)}_{k}$ and ${\left(q_{i}^{\prime }\right)}_{k}$. With the entropy inequality, now written in terms of ${\bar{w}}_{st}$ and ${\bar{q}}_{i}$, we get the formula below.

(26) $$\;-\left(\eta \dot{T}+\dot{\psi }\right)+{\bar{w}}_{st}-\frac{1}{T}{\bar{q}}_{i}T_{,i}\geq 0,$$

and by employing $\psi =\psi \left(v_{I},{\tilde{\zeta }}_{J},\;T\right)$, we find the following equation by using the Coleman-Noll procedure.

(27) $$\eta =-\frac{\partial \psi \left(v_{I},{\tilde{\zeta }}_{J},\;T\right)}{\partial T}.$$

Using these approaches, it can be demonstrated by employing different representations for $w_{st}^{\prime }$ and $q_{i}^{\prime }$ that Toupin-Mindlin type and non-Toupin-Mindlin type gradient elasticity models can be derived. For the former, the Cauchy stress $\Sigma_{jk}$ is given by the Euler-Lagrange derivatives.

(28) $$\Sigma_{jk}=\frac{\partial \psi }{\partial \varepsilon_{jk}}-\;{\left(\frac{\partial \psi }{\partial \varepsilon_{jk,i}}\right)}_{,i},$$

For the latter, we gain the equation below.

(29) $$\;\Sigma_{jk}=\frac{\partial \psi_{1}}{\partial \varepsilon_{jk}}$$

is obtained, which does not exhibit the Euler-Lagrange structure. For details and one-dimensional applications, the reader is referred to the paper.

Y. Hua, T. Zhao & Z. G. Z.-Y. Guo [56] “*Irreversibility and Action of the Heat Conduction Process*”. This article is the authors’ assertion that irreversibility of a physical process can be characterized by Lyapunov functions of stability theory. The authors express their conviction that irreversibility means that, in such a system, a Lyapunov function exists and that the equilibrium state is the attractor for non-equilibrium states. Accordingly, entropy and its production are regarded as Lyapunov functions of various irreversible processes. However, since, in the stability theory, the Lyapunov function is not always unique. It is inferred that, in the authors’ view, entropy does not need to be the vehicle to judge the irreversibility of the processes. They suggest *entransy* – a concept introduced by them in earlier papers to take the role of the Lyapunov function characterizing irreversibility and they demonstrate its “*workability*” in the principle of least action for heat conducting processes.

C. Miller, W. Gray & C. Kees [57] “*Thermodynamically Constrained Averaging Theory: Principles, Model Hierarchies, and Deviation Kinetic Energy Extensions*”. Macro-scale models in continuum physics are based on certain averaging procedures and the avoidance of resolving the morphology of the distribution of the phases at the micro-scale. Various procedures exist to derive macro-scale models based on micro-scale models, which resolve some of the subscale properties. The authors’ concept is called the thermodynamically constrained averaging theory (TCAT) and is an almost exclusive construct for achieving macro-scale modeling.

The article presents a detailed introduction into the concept and structure, how the upscaling from the micro-scale to the macro-scale is performed, and it demonstrates without digging into mathematics how the averaging process is incorporated. One important step is that the micro-scale is referred for each representative volume (surface, line) element to a localized coordinate. This prerequisite entails separating the field variables into global contributions, which is not dependent on the local variables and micro-fields. This depends on global and local coordinates and vary within the size of the representative volume (surface, line) element and whose averages of products determine the influence of the physics on the micro-scale to the macro-scale. This also gives rise of such terms on the macro-scale entropy inequality and all other macro-scale physical balances laws. A prominent example of this sort is rapid flow of debris, a granular-fluid mixture, in which the solid and the fluid particles perform averaged motions with solid and fluid fluctuation motions.

This concept, of course, entails construction of the entropy inequality at the macro-level, which needs to be constrained by the physical macro-field equations. This step is accounted for by the construction of the extended entropy inequality in which the macroscopic field quantities are incorporated by Lagrange parameters. The detailed calculations are explained by using the deviation kinetic energies of the phases and operating within the macro-field equations of mass, momentum for the phases, and their contact interfaces and contact lines. For readers not knowledgeable with such mathematical complexities, this part of the manuscript must be enigmatic. In such cases, the text by Gray & Miller [58] is recommended. This reference is also recommended for readers who are better equipped with multiphase models. It is not explained how the Lagrange parameters are determined and how, in detail, the constitutive restrictions of the non-equilibrium variables are obtained.

## Abbreviations

**Roman Symbols**	
ARMA	Archive for Rational Mechanics and Analysis
***D*** = $\frac{1}{2}\left(\mathrm{grad}\;v+\;{\left(\mathrm{grad}\;v\right)}^{T}\right)$	Stretching tensor
EEI	Extended Entropy Inequality
EP	Entropy production
*f*	Body force per unit mass
grad T	Temperature gradient
*p*	Pressure
*q*	Heat flux vector
*r*	Heat supply rate per unit mass
Re	Reynolds number
SL	Second Law
*s*	Entropy supply per unit mass
$s_{i}$	Supply rate density of the *i*-th physical balance law
	[$s_{i}=f$ or $s_{i}=r$ for momentum and internal energy]
*T*	Absolute temperature
*t*	Time variable
$w_{\mathrm{st}}$	Stress power
**Greek Symbols**	
γ	Entropy production per unit mass
ε	Internal energy density per unit mass
*η*	Entropy density per unit mass
*θ*	Empirical temperature
$\Lambda_{i}$	Lagrange par. of the i-th phys. Balance law
$\lambda_{i}$	Prop. factor of the i-th supply rate density $s_{i}$
*ρ*	Mass density
Σ	Summation sign
$\sigma ,{\;\sigma }^{E}$	Cauchy stress tensor, Extra stress tensor
ϕ	Entropy flux vector
*ψ*	Helmholtz free energy
**Miscellanea**	
$\frac{d}{\mathrm{dt}}(\cdot )=\;{\left(\cdot \right)}^{\cdot }$	$\frac{d}{\mathrm{dt}}(\cdot )=\;{\left(\cdot \right)}^{\cdot }$
$\frac{\partial }{\partial x}(\cdot )$	$\frac{\partial }{\partial x}(\cdot )$
*x*, *y*, *z*	*x*, *y*, *z*
